# Bedside Measurement of Volatile Organic Compounds in the Atmosphere of Neonatal Incubators Using Ion Mobility Spectrometry

**DOI:** 10.3389/fped.2019.00248

**Published:** 2019-06-18

**Authors:** Julia Steinbach, Sybelle Goedicke-Fritz, Erol Tutdibi, Regine Stutz, Elisabeth Kaiser, Sascha Meyer, Jörg Ingo Baumbach, Michael Zemlin

**Affiliations:** ^1^Department of Applied Chemistry, Reutlingen University, Reutlingen, Germany; ^2^Department of General Paediatrics and Neonatology, Saarland University Medical School, Homburg, Germany

**Keywords:** ion mobility spectrometry, neonatal incubator, preterm infant, volatile organic compound, breath analysis

## Abstract

**Background:** Early and non-invasive diagnosis of common diseases is of great importance in the care of preterm infants. We hypothesized that volatile organic compounds (VOC) can be successfully measured in the neonatal incubator atmosphere.

**Methods:** This is a feasibility study to investigate whether the discrimination of occupied and unoccupied neonatal incubators is possible by bedside measurement of volatile organic compounds (VOCs) on the neonatal intensive care unit. VOC profiles were measured in the incubator air using ion mobility spectrometry coupled to multi-capillary columns (BreathDiscovery B&S Analytik GmbH, Dortmund, Germany).

**Results:** Seventeen incubators occupied by preterm infants (50 measurements) and nine unoccupied neonatal incubators were sampled, using 37 room air measurements as controls. Three VOC signals that allow the discrimination between occupied and unoccupied incubators were identified. The best discrimination was reached by peak P20 exhibiting a sensitivity, specificity, positive predictive value and negative predictive value of 94.0, 88.9, 97.3, and 72.3%, respectively. Use of a decision tree improved these values to 100.0, 88.9, 98.0, and 100.0%, respectively.

**Discussion:** A bedside method that allows the characterization of VOC profiles in the neonatal incubator atmosphere using ion mobility spectrometry was established. Occupied and unoccupied incubators could be discriminated by characterizing VOC profiles. This technique has the potential to yield results within minutes. Thus, future studies are recommended to test the hypothesis that VOCs within neonatal incubators are useful biomarkers for non-invasive diagnostics in preterm neonates.

## Introduction

More than one in ten infants is born prematurely (1, 2). The risk of complications is inversely correlated with lower gestational age (3, 4). Common complications in preterm infants are sepsis, bronchopulmonary dysplasia (BPD), retinopathy of prematurity (ROP), intraventricular hemorrhage (IVH), and necrotizing enterocolitis (NEC) (1, 4–8). However, diagnosis is dependent on the combination of various symptoms and invasive methods like blood analysis (6–10). Non-invasive diagnosis via detection of volatile organic compounds (VOCs) has been examined for sepsis, NEC, and BPD in several studies with promising results (10–16). The most commonly used device is the electronic nose (eNose) that consists of an array of sensors, which allows to distinguish groups based on their specific so called “smell prints” (11–15). Nevertheless, this method of pattern recognition does not allow the characterization and identification of possible biomarkers. In addition, biological samples such as tracheal aspirate (12) and feces (11, 13–16) were used at defined time points. Exhaled air on the contrary is released with every breath and is therefore particularly suitable as a sample for non-invasive diagnosis and continuous monitoring. Ion mobility spectrometry (IMS), which allows the detection of VOCs in lower ppb_v_ to ppt_v_ (ng L^−1^ to pg L^−1^) range, has the potential to be used as a bedside method (17, 18).

IMS coupled to multi-capillary columns (MCC/IMS) have been successfully used for the non-invasive diagnosis of several diseases (19–22) and for the determination of characteristic VOCs and patterns from *Escherichia coli* (23, 24) and *Pseudomonas aeruginosa* (24, 25).

One major advantage of bedside IMS measurements is that results are available within minutes. However, multiple potential contaminants such as VOCs from hand sanitizers, plastic ware, and other materials must be considered to ensure the specificity of potential volatile biomarkers (26–28).

Therefore, the first step toward non-invasive diagnosis from neonatal incubator air is to establish a sampling technique for neonatal incubator air and to investigate whether distinction of occupied and unoccupied neonatal incubator can be achieved by single VOCs or groups of VOCs arising from the neonatal incubator air using MCC/IMS.

## Materials and Methods

This study was performed at the Department of Pediatrics, Saarland University Medical Center, Homburg (Germany) and was approved by the Ethics Committee Saarbrücken (reference 276/17). Parents' written consent was obtained prior to the measurements. Premature infants weighing <1.800 g that were treated in an incubator were enrolled in the study. The model of neonatal incubator was the THERMOCARE Vita (WY2402, Weyer GmbH, Kürten-Herweg, Germany).

For analysis of neonatal incubator air, a MCC/IMS BreathDiscovery (B&S Analytik GmbH, Dortmund, Germany), laptop computer and a synthetic air gas bottle were placed on a metal cart. The isothermal (40°C) pre-separation was accomplished with an OV-5 multi-capillary column (MCC) (Multichrom, Novosibirsk, Russia). The device and sampling parameters are given in Supplemental Table 1. The methods for VOC analysis were published earlier (17, 19–21, 29–31). All samples were taken with polytetrafluorethylene tubing covered by a single-use sterile glass pipette to avoid contamination between the neonatal incubators. The glass pipette was inserted with a length of proximately 10 cm through the side ports (grommets for tubes and cables of medical devices) at the head side of the neonatal incubators. According to pilot studies and the device standard, for MCC/IMS a sampling time of 20 s with a sample flow of 100 mL/min was selected. Isothermal separation was performed at 40°C with an MCC flow of 150 mL/min. Drift flow was set to 100 mL/min and the ions were detected in positive mode.

### Data Assignment

A total of 17 preterm infants were measured with a sample count of 50 measurements. Each measurement was a complete MCC/IMS chromatogram consisting of 1.500 single spectra with 2.500 data points per spectra. As a reference, nine unoccupied neonatal incubators were measured with an average temperature of 36°C (32–37°C) and an average relative humidity of 60% (55–67%) as well as 41 room air measurements.

**Figure 1:**
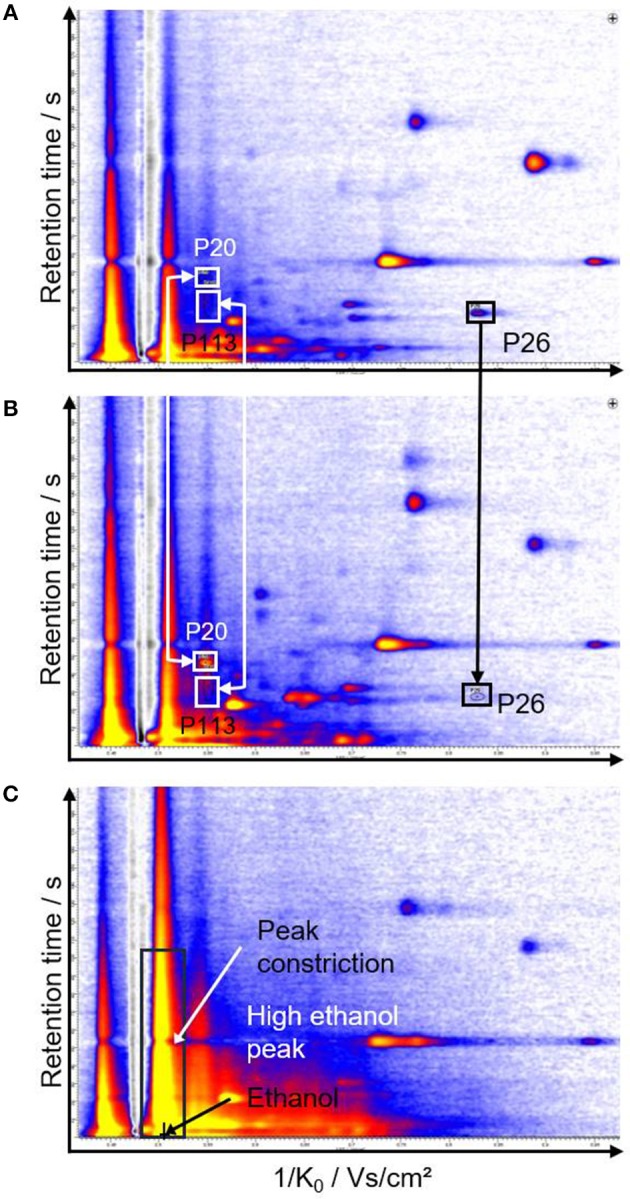
Representative chromatograms of unoccupied **(A)** and occupied incubators **(B)**. The peaks that allow discrimination of the groups are indicated with arrows and rectangles. **(C)** shows a chromatogram with a strong ethanol peak leading to peak constriction.

Fifteen measurements showed particularly high concentrations of ethanol that most likely originated from disinfectants. An overload was defined as the predominance of a given signal (ethanol) that overlaid neighboring peaks and that was associated with a peak constriction (Figure 1). To rule out a distortion of the analyses due to strong ethanol peaks, a two-step evaluation was chosen. First, the 15 overloaded measurements (4 room air samples and 11 preterm infant samples) were excluded for model generation (model data set). The model data set therefore included measurements of 39 preterm infants, 9 unoccupied neonatal incubators and 37 room air samples. In the second step, the primarily excluded overloaded measurements were reincluded into the dataset for model validation. The validation data set included 50 preterm infants, 9 unoccupied neonatal incubators, and 41 room air samples.

In contrast to occupied incubators, which exhibit a high natural variability in VOC patterns, unoccupied incubator VOC patterns are almost constant over time. To avoid statistically overestimated significance of the unoccupied incubator peaks, no replicate measurements were included in the evaluation.

### Statistical Analysis

The generated MCC/IMS data were evaluated with the software VisualNow 3.7 (B&S Analytik GmbH, Dortmund, Germany). All peaks were characterized by their specific combination of retention time per second and drift time (corresponding 1/K0-value). The peak height is correlated to the concentration (32). The databank layer 20160426_SubstanzDbNIST_122_St_layer (B&S Analytik GmbH, 2016) was used for peak referencing and determination of retention times and 1/K0-values. Box-and-Whisker plots and a rank sum test (Wilcoxon-Mann-Whitney test using Bonferroni correction) were used. Significant peaks [*p* < 0.05, 95% confidential interval (CI)] were used for further evaluation with decision trees (DT) (33, 34) using RapidMiner Studio Free 8.2.001 (RapidMiner GmbH, Dortmund, Germany) and principal component analysis (PCA) (35, 36) using Unscrambler X 10.4 (CAMO Software AS, Oslo, Norway).

## Results

A total of 149 signals (peaks) were detected from room air, neonatal incubators and preterm infants. A reduced dataset was generated that only includes signals with significance after Bonferroni correction of *p* < 0.05 (95% CI) for the separation of at least two groups. These 73 peaks were included for further evaluation. Representative chromatograms are shown in Figure 1.

For the distinction of neonatal incubators and preterm infants, three peaks with a significance of at least *p* < 0.05 (95% CI) after Bonferroni correction were found. The average (± standard deviation) signal intensities of P20 and P113 were higher for preterm infants (50.2 ± 23.2 V, 46.8 ± 26.3 V) than for room air (15.1 ± 5.3 V, 17.5 ± 7.4 V) and neonatal incubators (21.7 ± 5.2 V, 19.0 ± 6.7 V). For P113 (*p* < 0.05), P20 (*p* < 0.01), and P26 (*p* < 0.01) a sensitivity/specificity of 94.9%/55.6%, 92.3%/88.9%/, and 100.0%/55.6%, as well as positive/negative predictive values of 90.2%/71.4%, 97.3%/72.7%, and 90.7%/100.0% were determined, respectively (Table 1).

**Table 1:** Statistical analyses for the model set and validation set for peaks P20, P26, and P113.

**Separation PTI/NI**	**Model set/Validation set**											
**Signal**	**P20**			**P26**			**P113**			**Decision tree**		
Signal Intensity	PTI > NI			PTI < NI			PTI > NI			Distinction possible		
Significance^*^	<0.01	/	<0.01	<0.01	/	<0.05	<0.05	/	<0.01	n.a.		
Sensitivity/%	92.3	/	94.0	100.0	/	96.0	94.9	/	96.0	100.0	/	100.0
Specificity/%	88.9	/	88.9	55.6	/	55.6	55.6	/	55.6	100.0	/	88.9
PPV/%	97.3	/	97.3	90.7	/	92.3	90.2	/	92.3	100.0	/	98.0
NPV/%	72.7	/	72.2	100.0	/	71.1	71.4	/	71.4	100.0	/	100.0
Accuracy/%	91.7	/	93.2	91.7	/	89.8	87.5	/	89.8	100.0	/	98.3
PTI/RA Signal Intensity	PTI > RA			PTI > RA			PTI > RA			Distinction possible		
Significance^*^	<0.001	/	<0.001	<0.001	/	<0.001	<0.001	/	<0.001	n.a.
Sensitivity/%	92.3	/	94.0	88.9	/	96.0	89.7	/	90.0	100.0	/	98.0
Specificity/%	94.6	/	92.7	100.0	/	70.7	86.5	/	87.8	91.9	/	92.7
PPV/%	94.7	/	94.0	100.0	/	80.0	87.5	/	90.0	92.9	/	94.2
NPV/%	92.1	/	92.7	97.4	/	93.5	88.9	/	87.8	100.0	/	97.4
Accuracy/%	93.4	/	93.4	97.8	/	84.6	88.2	/	89.0	96.1	/	95.6
NI/RA Signal Intensity	NI > RA			NI > RA			NI < RA			Distinction possible		
Significance^*^	n.a.	/	n.a.	<0.001		<0.001	n.a.	/	n.a.	n.a.
Sensitivity/%	66.7	/	66.7	88.9	/	88.9	0.00	/	0.00	88.9	/	100.0
Specificity/%	89.2	/	87.8	100.0	/	97.6	100.0	/	100.0	100.0	/	100.0
PPV/%	60.0	/	54.5	100.0	/	88.9	n.a.	/	n.a.	100.0	/	100.0
NPV/%	91.7	/	92.3	97.40	/	97.6	80.4	/	82.0	97.4	/	100.0
Accuracy/%	84.8	/	84.0	97.8	/	96.0	80.4	/	82.0	97.8	/	100.0

*PTI, preterm infant; NI, neonatal incubator; RA, Room air; PPV, positive predictive value; NPV, negative predictive value; x, significant separation; –, no significant separation*.

* *Significance after Bonferroni correction*.

**Figure 2:**
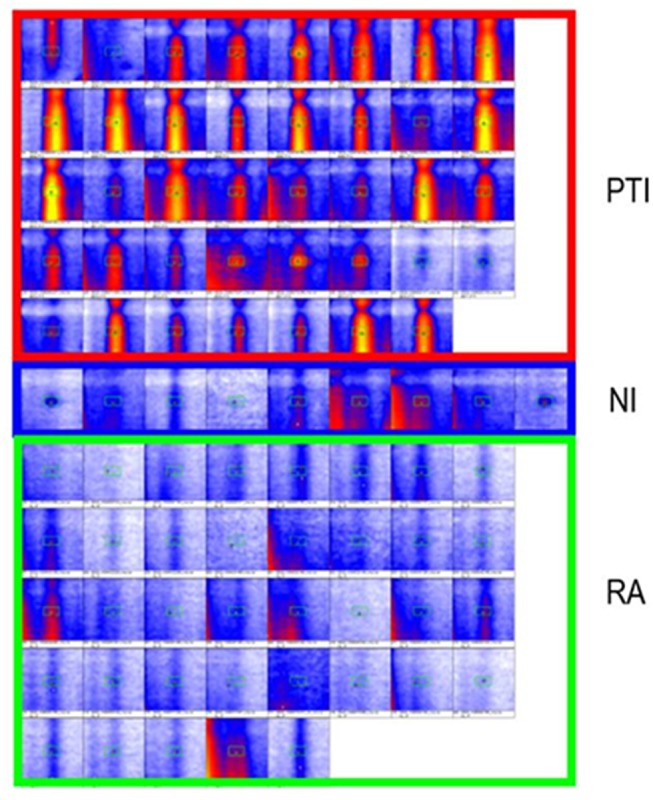
Intensity distribution of P20. The best threshold for the separation (26 mV) is displayed. PTI, preterm neonate infant; NI, neonatal incubator; RA, Room air.

**Figure 3:**
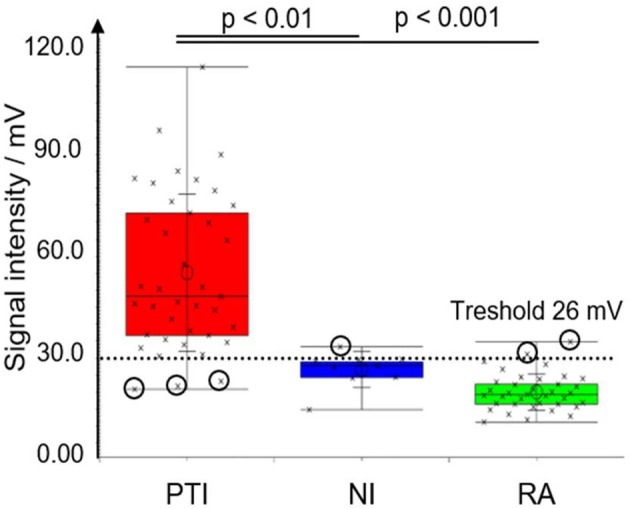
Box-and-Whisker plot for peak P20 for original data set (n_NI_ = 9, n_PTI_ = 39, n_RA_ = 37). Measurements are marked with crosses; incorrectly classified measurements are encircled. Significance levels after Bonferroni correction are indicated. PTI, preterm neonate infant; NI, neonatal incubator; RA, Room air.

The peak area for P20 differed significantly between preterm infants vs. unoccupied neonatal incubators and room air, respectively (Figure 2). For P26 unoccupied neonatal incubators showed the highest intensity with 13.2 ± 6.9 V in comparison with 5.5 ± 2.0 V and 3.0 ± 1.4 V for preterm infants and room air, respectively (Figure 3).

All 73 significant peaks were analyzed by PCA with 5 principal components (PC) using the peak heights as a variable and each peak as a sample (Supplemental Figure 1). Using 5 principal components, the explained variance was 88.3% for the distinction of all three groups and 85% for the distinction of preterm infants and unoccupied neonatal incubators. This PCA did not allow a clear distinction between the measurement from preterm infants and unoccupied neonatal incubators (Supplemental Figure 1).

The method of calculating decision trees confirmed in the model data set and in the validation data set that the peaks P20, P26, and P113 that had been previously identified by Bonferroni correction were best suited to differentiate between preterm neonates, unoccupied neonatal incubators and room air (Supplemental Figures 2, 3).

In the model data set the decision tree reached a 100% separation of preterm infants and unoccupied neonatal incubators using peaks P20, P26, and P113 (Table 1). Moreover, the sensitivity, specificity, positive predictive value and negative predictive value all reached 100%. In the validation data set, the sensitivity, specificity, positive predictive value and negative predictive value were 100, 88.9, 100, and 98.3%. For both data sets, the peak P113 was not required to distinguish the groups via decision tree analysis.

## Discussion

We demonstrate the feasibility of a novel technique that allows bedside measurements of VOC profiles using ion mobility spectrometry of the neonatal incubator atmosphere. Using this method, it was possible to distinguish VOC profiles of neonatal incubators that were occupied with preterm neonates from unoccupied neonatal incubators.

Most of the currently used biochemical diagnostic tests are based on non-volatile molecules that are solved in body fluids such as serum, plasma, saliva or urine. In particular, blood tests which are routinely required in the treatment of preterm neonates, are associated with distress during blood sampling, increased need for blood transfusion and time delays during sample processing. However, it is known that volatile organic compounds are released during many biological pathways and can serve as biomarkers in adults (37), children (38), and neonates (11–16). Previous studies usually used exhaled breath (19, 39) or headspace measurements of biosamples (11–16).

In this study it was confirmed that the presence of a preterm neonate is associated with changes in the VOC profile within the neonatal incubator atmosphere, using room air samples as controls. The presence of a preterm neonate was associated with changes in the VOC profile within the neonatal incubator atmosphere, using room air samples as controls. In this feasibility study 73 signals from VOCs were identified that differed significantly after Bonferroni correction between preterm infants, unoccupied neonatal incubators and room air. PCA of these 73 signals was insufficient to reliably differentiate the three groups of samples. This finding was expected since the majority of VOCs in all three groups most likely originated from environmental sources such as room air and equipment. Moreover, the number of measurements was relatively low (*n* = 100 measurements).

However, statistical analyses on individual peaks and on groups of peaks, including the calculation of decision trees, was successfully used to identify three peaks that allowed the distinction of occupied and unoccupied neonatal incubators. The biochemical identification of the VOCs responsible for peaks P20, P26, and P113 is part of ongoing experiments. The concentration of these analytes was too low for identification by gas chromatography/mass spectrometry (GC/MS). Therefore, sample enrichment is required in further experiments since the detection limit for MCC/IMS is lower compared to GC/MS analysis. However, a clear, reproducible identification of the peaks is provided by the specific drift and retention time, irrespective of the chemical identification of the molecule.

It was found that even the predominance of one particular environmental peak, ethanol, did not impede the analyses in our setting since the model data set (excluding measurements overloaded with ethanol) and the validation data set (including overloaded measurements) yielded similar significances for the diagnostically relevant peaks P20, P26, and P113 in this series.

However, there is a high risk that ethanol and other environmental VOCs may disturb the detection of VOCs that would be important for diagnostic use, including inflammatory diseases such as sepsis, BPD and NEC. For example, Fink et al. (40) found 3-pentanone and acetone to be significant for the distinction of sepsis vs. healthy control group in a rat model. Notably, 3-pentanone (1/K0 = 0.546 Vs/cm^2^; RT = 7.4 s) and acetone (1/K0 = 0.544 Vs/cm^2^, RT = 2.4 s) both eluate in the proximity of ethanol (1/K0 = 0.510 Vs/cm^2^, RT = 0.0 s) and therefore might be hidden at high ethanol concentrations. Hsieh et al. (27) demonstrated that the ethanol concentration in neonatal incubators remained in a concentration of up to 5 ppm_v_ 30 min after the use of hand sanitizers. Ulanowska et al. (28) reported a calibration range for MCC/IMS of ethanol in the ppb_v_ range, which is approximately 1/100–1/1,000 of the expected concentration according to Hsieh et al. (27).

Thus, the excessive sensitivity of IMS measurements and the highly variable environmental load with VOCs remain the crucial factors that need to be considered when volatile biomarkers are to be identified for diagnostic use. Potential approaches to reduce the effects of disinfectants and other potential contaminants on the measurements could be modifications of the multi-capillary flow column, or a reduced sampling time. A mathematical exclusion of the ethanol peak is also conceivable. All empty incubators were prepared and equipped identically to the occupied incubators with the only difference of the infant being present or not. Thus, we conclude that peaks P20, P26, and P113 are highly likely to be associated with the infant. Moreover, peaks P20, P26, and P113 were not detectable in potentially contaminating environmental sources such as diapers, clothing, pacifiers, medical devices, and skin care products (data not shown).

Taken together, the established method of bedside IMS measurements coupled to a multi-capillary column and the results of the presented feasibility study can be useful for the future development of non-invasive diagnostic methods using VOC analysis in the incubator atmosphere of preterm infants.

## Data Availability

The datasets supporting the conclusions of this article are included within the article or are available from the authors upon request.

## Ethics Statement

After written informed consent, 30 mL air were sampled from the incubator atmosphere. No additional interaction was performed. This study was approved by the Ethics Committee of the Philipps-University Marburg. AZ 91/11.

## Author Contributions

MZ, SM, ET, SG-F, and JB designed the study. EK and RS supported organisational and coordinative tasks. SG-F and JS performed the experiments. JS evaluated the data. All authors contributed to writing the manuscript.

### Conflict of Interest Statement

Since September 2018 JB is an employee of a company producing spectrometers as used in the present paper. The remaining authors declare that the research was conducted in the absence of any commercial or financial relationships that could be construed as a potential conflict of interest.

## Abbreviations

CI: Confidential interval
DT: Decision tree
IMS: Ion mobility spectrometry
IVH: Intraventricular hemorrhage
MCC: Multi-capillary column
MCC/IMS: Ion mobility spectrometer coupled to multi-capillary column
NI: Neonatal incubators
PCA: Principal Component Analysis
PTI: Preterm infant
R: Room air
ROP: Retinopathy of Prematurity
TDS-GC/MS: Thermo-desorption-gas chromatography coupled to mass spectrometry
VOC: Volatile organic compound.
