# Supplementary material for: Bedside Measurement of Volatile Organic Compounds in the Atmosphere of Neonatal Incubators Using Ion Mobility Spectrometry
Source: Front Pediatr. 2019 Jun 18;7:248. doi: 10.3389/fped.2019.00248 (PMC6591267; doi:10.3389/fped.2019.00248)
Supplement: Supplementary file 1 [file Data_Sheet_1.pdf]

Bedside measurement of volatile organic compounds in the atmosphere of neonatal incubators using ion mobility spectrometry

Julia Steinbach, Sybelle Goedicke-Fritz, Erol Tutdibi, Regine Stutz, Elisabeth Kaiser, Sascha Meyer, Jörg Ingo Baumbach, Michael Zemlin

Supplemental Figures and Table

Supplemental
FIGURE 1

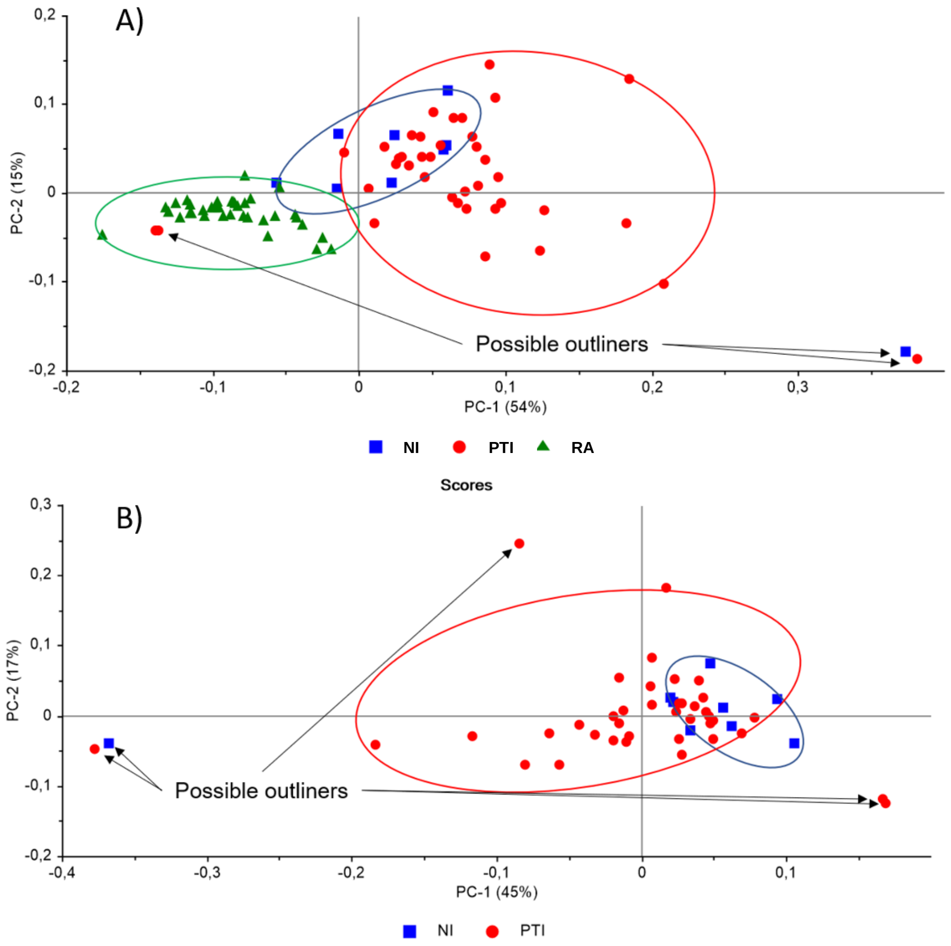

Supplemental Fig. 1. A) PCA (PC-1 – PC-2) of 73 significant peaks. Measurements were grouped according to their classes (blue = NI neonatal incubators, red = PTI, preterm infants, green = RA, room air). Possible outliners have been marked. B) PCA (PC-1 – PC-2) of PTI (red) and NI (blue). The NI group strongly overlaps with the PTI group. Possible outliners have been marked.)

Supplemental
FIGURE 2

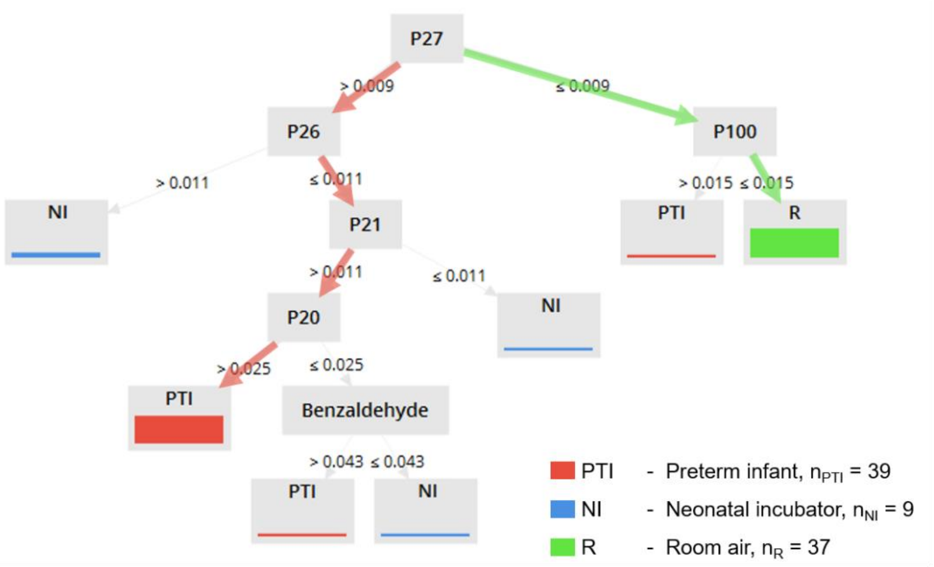

Supplemental Fig. 2. Decision tree (DT) for significant peaks. Discrimination of all three groups is possible. Every measurement was correctly assigned to its class. The main paths are colorized. Benzaldehyde was identified via comparison of retention and drift times with measurements of a calibration mixture containing benzaldehyde.

Supplemental
FIGURE 3

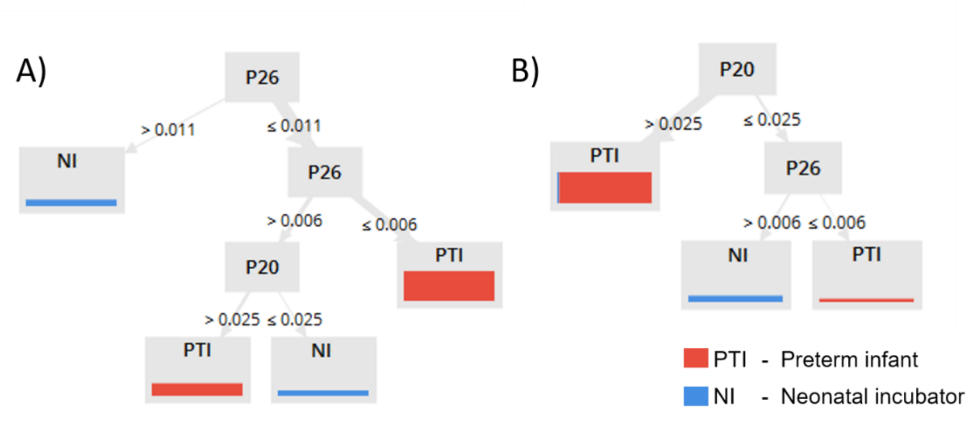

Supplemental Fig. 3. DT with significant peaks P113, P20 and P26 for A) model set (n_{NI} = 9, n_{PTI} = 39) without overloaded measurements and B) validation set (n_{NI} = 9, n_{PTI} = 50) including primarily excluded overloaded measurements for validation. Sensitivity/specificity was 100.0 %/100.0 % for A) and 100.0 %/88.8 % for B) with positive prediction value/negative prediction value 100.0 %/100.0 % for A) and 98.0 %/100.0 % for B).

Supplemental
TABLE 1

Supplemental Table 1. Device and sampling parameters of MCC/IMS

	**MCC/IMS**
**Column**	OV-5
**Column temperature**	40 °C
**Sampling type**	Direct
**Sampling duration**	20 s
**Sample flow**	100 mL/min
**MCC flow**	150 mL/min
**Drift flow**	100 mL/min
**Carrier gas**	Synthetic air (20.5 ± 0.5% O_2 in N_2, purity ≥ 99,999 mol%)
**Ionisation**	^{63}Ni (95 MBq)
**Ion separation**	Drift tube (330 V/cm, 0.12 m)
**Detection mode**	positive
